# High blood pressure inhibits cardiovascular responsiveness to expressive classical music

**DOI:** 10.1038/s41598-025-94341-2

**Published:** 2025-03-29

**Authors:** Vanessa C. Pope, Mateusz Soliński, Pier D. Lambiase, Elaine Chew

**Affiliations:** 1 Faculty of Life Sciences & Medicine, School of Biomedical Engineering & Imaging Sciences, King’s College London, 1 Lambeth Palace Rd, London, SE1 7EU UK; 2 Faculty of Natural, Mathematical & Engineering Sciences, Department of Engineering, King’s College London, Strand, London, WC2R 2LS UK; 3 Barts Heart Centre, St Bartholomew’s Hospital, West Smithfield, London, EC1A 7BE UK

**Keywords:** Hypertension, Blood pressure, Music, Variability, Physiology, Tempo, Loudness, Hypertension, Human behaviour

## Abstract

Music lowers hypertensive patients’ blood pressure (BP) in the long-term, but the dynamics of BP during music-listening are not well understood. This study aims to determine: (1) whether individuals with high and normal BP respond to music differently; and, (2) whether music’s loudness or tempo drives these differences. Music with computer-altered tempo and loudness is rendered on a reproducing piano to 40 middle-aged participants, 20 with baseline BP above 140/90 mmHg (H-bBP) and 20 below (N-bBP) but above 90/60 mmHg, paired by playlist. Continuous BP was recorded whilst they listened to playlists of 9 tempo- and loudness-transposed versions of 8 distinct pieces of Western classical music (40 min) after a 5-minute baseline silence. Both participant groups’ mean systolic and diastolic BP rose significantly higher than baseline during music listening, with normotensives’ mean systolic and diastolic BP rising significantly more than hypertensives’. Both groups’ BP variability (indexed by range and standard deviation of continuous BP measurements) reduced during faster tempi, but not during increased loudness. BP variability is significantly higher for both groups during the slowest pieces, which maintain the originally performed tempi. These findings suggest that music’s long-term benefit, like exercise, may come from its power to temporarily physiologically activate listeners.

## Introduction

Variability is a desirable feature in many physiological parameters: optimal physiological function is flexible and responsive to environmental and internal changes. Rigidity in a physiological signal, from heart rate variability (HRV) to alpha brain waves, is usually associated with negative health outcomes^1–3^. Heart rate variability is a valuable indicator of cardiac health, reflecting both the state of the autonomic nervous system (ANS) and the flexibility of cardiovascular mechanics, such as arterial stiffness. The ANS has a key role in regulating BP, particularly the fight-or-flight sympathetic drive, while BP levels in turn impact the ANS in a complex loop^4^. However, large increases in BP variability (BPV), often measured over hours, days, months or years, are associated with negative long-term outcomes^5–9^, as increases of variability may indicate the BP-ANS loop isn’t functioning well enough to keep BP in a healthy range.

Hypertension was estimated to affect 31.1% of the global population in 2010, and its prevalence is projected to rise^10^. While some hypertension medication operates on the mechanics of high BP by relaxing arteries or removing excess water from the blood, many hypertension medications intervene directly on the ANS and central nervous system by inhibiting hormones associated with the contraction of blood vessels^11^. While the risks associated with hypertension outweigh the side-effects of medications, non-pharmacological approaches to treating hypertension are desirable for all patients given the complex functions of the ANS, and crucial for the estimated 10-14.7% of patients with treatment-resistant hypertension^12^.

In the medium to long-term, music-listening has been found to lower both systolic blood pressure (SBP)^13–15^, diastolic blood pressure (DBP)^16,17^, or both^18–21^. Some participatory music interventions also include activities that independently impact cardiovascular function by controlling and slowing respiration, such as singing^22^ or wind instrument-playing^23.^. In this article, we will focus on isolating the effect of music-listening. In most music-listening studies, while a piece may be named, descriptions and measurements of musical features are not fully specified. Instead, the music is characterised more generally with terms like “relaxing.” Western classical music, despite its stylistic diversity, is often conflated with music that has a tranquilising effect: “classical music” is the most frequently used music genre in studies of music’s effect on anxiety^24.^. The physiological expectation for tranquilising music is that it reduces stress and sympathetic activity, lowering BP, heart-rate and increasing heart-rate variability^25^. A recording of music by Bach (classical) was found to lower SBP, DBP and HR more than that by the band Disturbed (heavy metal) in healthy humans and young pigs^26^. Pieces by Mozart and Strauss lowered BP more than uniform pop music^27^, and changes in music phrases have been linked to acute changes in skin vasomotion^28–30^. Pieces by Vivaldi and Bach were included in a selection of ‘pleasant music’ pieces that were found to raise heart rate and sympathetic activity^31^. The inconsistency of findings in music-based interventions, highlighted in meta-analyses, may be due in part to a lack of systematic study of the impact of specific musical features on physiology^13,32–35^.

When considering music features, studies of tempo’s physiological effect have used a range of stimuli: isochronous pulses^31^, beats in ascending, descending or stable tempi^36^, recorded music selected for its tempo^28,37^, algorithmic music^38^ and manipulated music^31^. Studies of the physiological effect of musical features can conflate tempo with genre by selecting music of different tempi from different genres, for example selecting a slow classical piece and a fast rap piece^39^. Fast tempo music is generally associated with an acute rise in blood pressure^37,40^, though findings are inconsistent: listeners of faster pieces showed a drop in BP rather than a rise in at least two studies^18,31^. Using genre as an index for tempo introduces a range of confounding variables. For example, some genres of music, such as pop or operatic arias, typically include singing and lyrics, while others, such as Western classical music, do not. Differences in response to a fast pop song and a slow classical piece could be due to tempo, as intended, or because singing activates the body differently or even because of the lyrical content. Each genre carries its own cultural associations (e.g. “pop music is for dancing”, “classical music is relaxing”) and structural expectations (e.g. the chorus will return after the verse). In turn, each composition plays with these meanings and expectations to expressively communicate and connect with the listener. Where loudness is an independent variable, there is also a genre switch with implied loudness differences (e.g. quieter baroque music, louder heavy metal)^41^. While it is possible to have loud baroque music and quiet heavy metal, genres were chosen for their typically divergent loudness profiles and no within-genre comparisons were made. There are few studies into the effect of loudness with expressive music, and none that we know of that examines BP specifically.

Stripping music back to its constituent parts – rhythmic patterns, sine tones, sound frequencies – and studying each variable independently is a tempting scientific approach that removes confounding variables. However, music’s physiological impact may well be due to the intentional, expressive and aesthetic interactions between features. In one study, music generated algorithmically by manipulating parameters such as timbre, tempo and note density to meet therapeutic goals with no consideration for aesthetic value did not affect participants’ BP, though it did slow their heart rates^38^.

To generate musical stimuli to help meet therapeutic goals, we need a better understanding of how each feature impacts physiology within an experimental framework that allows music to be fully expressive, variable and ecologically-valid. Variability in music may support physiological responsiveness, which can only be assessed by systematically and continuously measuring music features in expressively varying music alongside physiological measures. In the research presented here, the effects of tempo and loudness are isolated by computationally transposing solo classical performances by world-class pianists to create fast, slow, loud and quiet [{fast/slow} $\times$ {loud/quiet}] versions of expressive music pieces. By varying only tempo and loudness, we control for the impact of other expressive musical features such as genre, musical structure, proportionate inter-onset-intervals, key and complexity, to name a few (see Method for details). Music impacts the body, but how does each musical feature impact the body during listening? The aim of this study is to consider how the metrics and variation of music features might impact blood pressure and blood pressure variability, and how responsiveness to music is mediated by baseline blood pressure in the hypertensive range.

## Method

### Experimental set-up

Continuous BP waveforms (CNAP) were recorded in the lab while participants were seated. This study is part of a larger project and the analyses presented here focus solely on BP measurements, although ECG, respiration and qualitative responses were collected during the experiment.

Each participant listened to a playlist of 9 reshaped versions of 8 distinct pieces (approx. 40 min) after a 5-minute baseline silence. Each participant group heard the same randomly selected 20 playlists (see Music Stimuli below). Music was played to participants on a reproducing grand piano (Bösendorfer VC280 Enspire PRO), able to reproduce each piece exactly with performance-quality acoustics. Two experimenters were present during the study. Between each piece, participants were asked about their experience of the music and there were no other pauses in the listening task. The study protocol conforms to the ethical guidelines of the 1975 Declaration of Helsinki and was approved by the Oxford C Research Ethics Committee of the UK Health Research Authorities (IRAS 242471) and the Research Ethics Office at King’s College London (minimal risk registration number: MRPP-22/23-34904).

### Participants

Forty participants (23 females, mean age of 43.46 (95 CI: 39.05-47.90)), 20 with baseline BP measurement above 140/90 mmHg (H-bBP) and 20 with bBP below (N-bBP), were paired by playlist. The exclusion criteria were beta-blocker intake or hearing impairment. Beta-blockers were excluded on the basis they are known to attenuate autonomic response. Participants on other medications took part to ensure we included older and medically-hypertensive participants, groups who are more likely to be medicated.

Participants were asked to refrain from caffeine, alcohol, stimulants, and heavy exercise prior to the study. Participants completed a questionnaire regarding their health, anthropometrics, ethnicity and musical experience prior to the study. Four participants did not disclose their weight and height, seven did not disclose their ethnicity.

N-bBP participants’ average systolic BP was 123.0 mmHg (95%CI: 118.85-127.15) compared to H-bBP participants’ average systolic BP of 148.25 mmHg (141.59-154.91). Average diastolic BP was 78.35 (75.21-81.49) for N-bBP participants and 94.5 mmHg (91.39-97.61) for H-bBP participants. Study participants were predominantly middle-aged (43.46 years (39.05-47.90)) with self-reported weight and height in an expected range for the UK (73.90kg (68.60-79.21); 170.23cm (166.90-173.56)). The two participant groups did not differ significantly in age, weight or height.

The average score on a subset of the Gold MSI musical sophistication score questionnaire was 4.51 out of 7 (4.14 - 4.87) and the two groups’ scores did not significantly differ. Participants were asked about their music listening habits, musical training and musical preferences. Ten percent of participants in each group selected “Classical Music” as the genre they listened to most, while 45% of all participants selected “Rock/Pop.” While overall music sophistication scores did not differ between groups, there were differences in the proportion of highly-trained musicians: 25% of H-bBP participants had more than 4 years of formal music training, compared to 5% of N-bBP participants. The two groups reported similar levels of no musical training: 50% of N-bBP participants compared to 40% of H-bBP participants.

Across both groups, 57.5% (H-bBP 50%, N-bBP 65%) of participants identified themselves as White, 17.5% did not state their ethnicity, 12.5% identified as Black or Black British, 7.5% as Mixed Ethnicity and 5% as Asian or Asian British.

### Physiological measurement and blood pressure variability indices

Continuous blood pressure was captured using a CNAP monitor and finger cuff, while participants had their arms in a sling to support their wrist at heart level. Respiration and ECG measurement from a Polar strap were collected via Bluetooth and synchronised via the HeartFM data-gathering app. After segmentation by musical piece, continuous systolic and diastolic BP values for each listen were normalised by subtracting the mean baseline values. The difference between each participants’ own baseline and their measures during music are analysed in this study, acknowledging individual differences in baseline physiology.

Standard deviation and range were selected as indices for BP variability analysis. Standard deviation of BP is the most commonly used variability index^42^, though it is not expressed proportionally to mean BP, limiting its descriptiveness in real-world settings where there are no baseline measurements and timescales are significantly longer. However, we calculated standard deviation based on baseline-normalised data for each participant, thereby taking an individual’s mean BP into account. There are other existing measures that are not used here.

### Music stimuli

A selection of eight Western classical music pieces were computationally altered to have faster tempi, higher loudness, or both, resulting in a total of 30 possible pieces (7 pieces $\times$ 4 versions + 1 piece $\times$focus on isolating the effect of music-listening. In 2 versions) used to create 40 playlists. The pieces were selected from the Bösendorfer Legendary Artists Collection (famous works performed by renowned pianists) (Table 1). The last author, who in addition to being an engineer is a concert pianist expercienced in adjudicating the musicality of machine-generated music, selected the pieces and worked with a professional sound designer to create the dataset. Classical piano music pieces were chosen for the study as they have wide stylistic range and musical complexity, but can be represented well with data and therefore played acoustically ‘live’ on a reproducing piano.

Seven of the pieces tolerated manipulations of both tempo and loudness without compromising musical coherence, while one piece only tolerated an alteration of tempo due to its large fluctuations in loudness dynamics. For all but one piece, Version 1 (V1) is the original version. Because in 7 out of 8 cases tempo was sped up, there is considerable overlap between original versions of a piece and its slowest version. V1/V2 are the slower, and V1/V3 the quieter, versions. V4 is both faster and louder. To create versions, the tempo and/or loudness of the piece were linearly altered, with manual adjustments if required to make the piece mechanically playable on the reproducing piano.

*La Cathédrale Engloutie* by Debussy was included in the dataset for its complexity, but had to be altered differently to retain musical coherence. The piece was made quieter (rather than louder); V2 is its source, and is used as the ‘normal’ version when comparisons are made between original and altered pieces. At raised loudness, the tempo could not be pushed as high as at softer volume. As the only piece where Version 3 has a faster tempo than Version 4 (Table 1), these versions are excluded from analyses that compare versions to one another.

The playlists were generated by randomising first the piece order, then the version. Each playlist ended with a repetition of the first randomly selected piece, but a different version. Playlists varied in length from 29-40 minutes. Twenty randomly selected playlists were listened to by both H-bBP and N-bBP participants.

Loudness feature summary statistics were calculated from continuous raw loudness, extracted from audio signals (.wav) recorded in situ using the python package cosmodoit^43^. As loudness was calculated from audio, increases in tempo caused increases in loudness: more notes played in quick succession leads to greater accumulation of sound waves in a given window. Tempo feature summary statistics were calculated from beat annotations generated through cosmodoit, which calls Nakamura’s algorithm^44^ to create an initial alignment, before being manually checked to ensure accuracy. Because tempo alterations were made at the level of Musical Instrument Digital Interface (MIDI) files rather than on audio, tempo alteration only affected the onset times of key strikes and not pitch. Pieces with faster tempo were shorter than the original pieces.

### Musical feature tertiles

Tempo and loudness levels in music can be summarised in different ways, each of which could be a candidate for triggering physiological response. Pieces were separated into three groups based on each feature’s average value, but also its standard deviation (an index of its general variability) and range (an index of how large variation is).

Music pieces were split by average tempo based on what would be considered low (slow, < 60bpm) and high bpm (fast, > 120 bpm), resulting in uneven track group sizes (slow average bpm: 12; medium average bpm: 8; fast average bpm: 10). Pieces were split into equal groups of 10 for standard deviations (group thresholds: < 11, 11-27.7; > 27.7 bpm) and maxima ( < 87, 87-210, > 210 bpm).

Music pieces were split into equally-sized groups of 10 by loudness in sones. A sone is a unit of measurement that takes into account the perceptual experience of loudness and can be converted to dB: $dBA = 33.2 * LOG10(Sones) + 28$. Summary statistics of loudness averages (tertiles: < 12.72 sones, 12.72-16.48 sones, > 16.48 sones), standard deviation (tertiles: < 4.72 sones, 4.72 - 6 sones, > 6 sones) and maxima (tertiles: < 37.5 sones, 37.5 - 47 sones, > 47 sones). Choice of summary statistic did not impact track groups for tempo very much, as pieces predominantly overlapped within each tempi tertile regardless of choice of summary statistic. However, choice of summary statistic for loudness altered the included pieces appreciably as there was less internal consistency between loudness features (Table 2). Tempo feature indices had more overlap than loudness indices. Choice of index (average, standard deviation or range) has more of an impact on the pieces included in loudness tertiles than it in tempo tertiles.

**Table 1:** Piece, performer and composer for each track, alongside average tempo and loudness for each version: V1 and V2 have the original tempo, V1 and V3 original loudness, V3 and V4 have increased tempo, V2 and V4 increased loudness.

Piece	Performer	Composer	V1 (ORIG.)		V2 (LOUDER)		V3 (FASTER)		V4 (BOTH)	
			BPM	Sones	BPM	Sones	BPM	Sones	BPM	Sones
Gavotte Op. 12 No. 2	Prokofiev	Prokofiev	110.58	15.05	110.68	20.54	199.05	17.39	199.02	23.84
“Ständchen” (Serenade)	Bauer	Schubert-Liszt	54.75	12.00	54.75	18.08	120.26	14.33	120.20	21.30
Piano Sonata No. 18 in D major, K.576 (1789) (Adagio)	Landowska	Mozart	46.45	12.14	46.45	16.36	92.62	14.23	92.86	20.12
La Cathédrale Engloutie (Sunken Cathedral)	Debussy	Debussy	38.66	7.00	**ORIG** 38.71	**ORIG** 12.27	134.97^†^	9.60^†^	96.64^†^	15.79^†^
Nocturne in F-sharp minor, Op. 48 No. 2	Hofmann	Chopin	68.85	13.49	*NA*	*NA*	172.10	16.46	*NA*	*NA*
Berceuse Op. 57 D-flat major	Reisenaur	Chopin	27.44	7.85	27.42	17.37	54.85	9.41	54.85	19.50
Sonata No. 14 in C-sharp minor, Op. 27 No. 2, “Moonlight”	Lhevinne	Beethoven	45.62	8.92	45.53	13.60	172.88	12.96	172.83	19.45
Ave Maria (originally published in 1853 as “Méditation sur le Premier Prélude de Piano de S. Bach”)	Lemer	Bach-Gounod	60.22	9.52	60.08	14.62	150.06	11.77	150.09	16.53

Debussy’s *Sunken Cathedral* is an exception, with V2 having the original loudness. There are only two versions of Chopin’s *Nocturne* as it was not perceptually appropriate at a louder volume. Small variations within versions with the same feature levels are due to manual adjustments to make a piece mechanically playable, or because interactions between features affect feature extraction. ^†^indicates data for Versions 3 and 4 of *Sunken Cathedral* excluded from version comparisons.

**Table 2:**
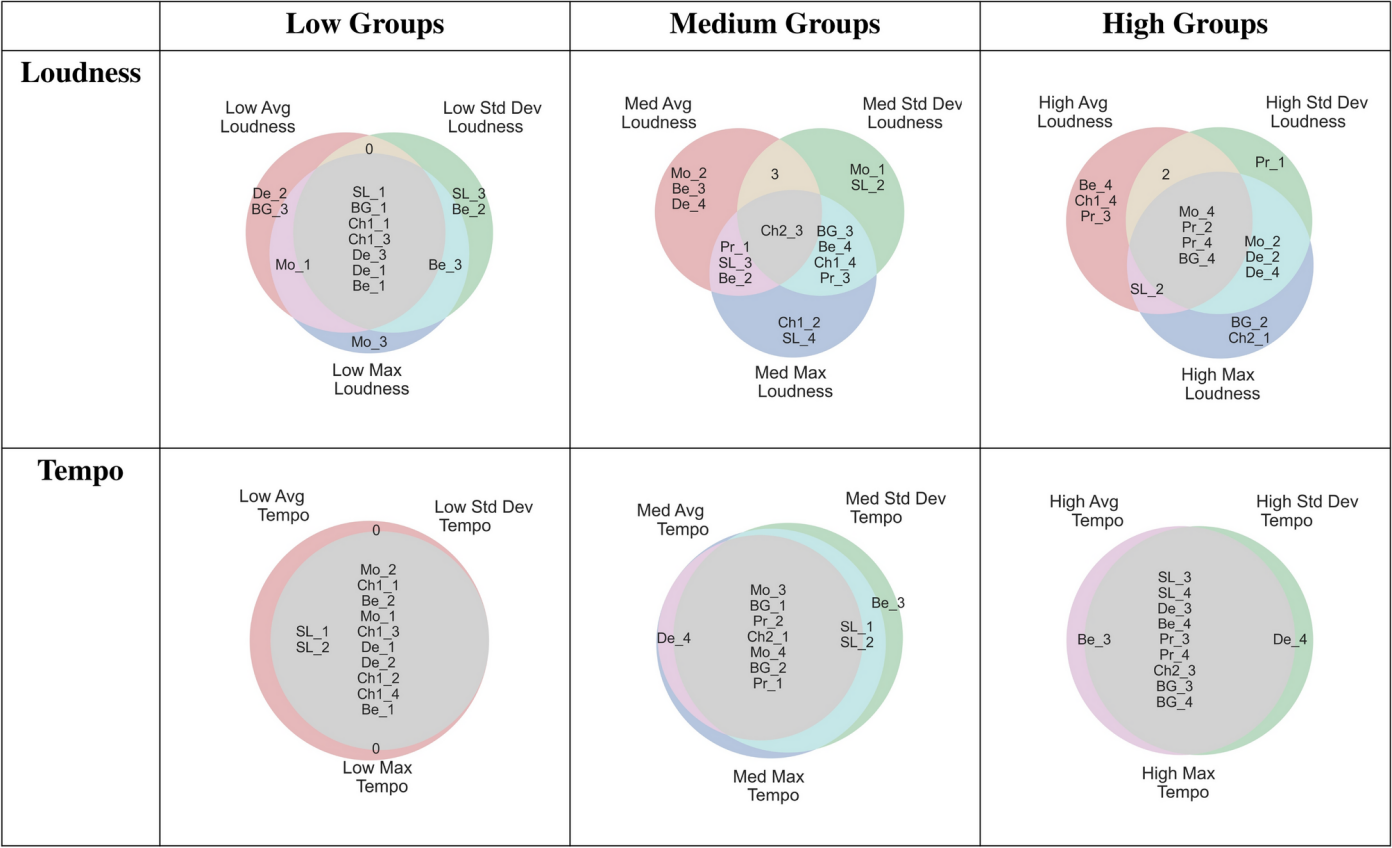
Tempo features are much more internally consistent than Loudness features, with most pieces overlapping no matter which features is used to divide pieces. Choice of loudness feature alters track groupings more dramatically, though low standard deviation, average and maximum groups overlap the most.

### Statistical methods

Using a Bonferroni correction to account for our analysis of six variables, we used a significance threshold of $\alpha = 0.0083$. In Tables, results < 0.0083 are indicated with **. All between-group tests were done using a Mann-Whitney U test for non-parametric data. Statistical testing was carried out in Python using the stats module.

A Kruskal-Wallis test is used for within-group tests, followed by a Dunn Test for post-hoc analysis of significant differences. If post-hoc analysis did not identify significant differences between specific groups, the Kruskal-Wallis test is treated as insignificant even if it is below the significance threshold. To safeguard against random effects, surrogate data was generated for each test by randomly re-allocating data into the appropriate number and size of sub-groups for that particular test over 1000 iterations. For example, a between-group test would create two randomly sampled datasets from across both groups 1000 times, then test for significance. A within-group test for versions would create four datasets with data drawn from all versions. These datasets were tested for statistical significance using the same test as the original comparison. All findings reported in this paper found no significant differences between randomly allocated surrogate data groups.

Prescriptive analyses were the comparison of BP variables between N-bBP and H-bBP groups. Exploratory analyses considered the impact of summary statistic groupings (averages, standard deviations and maxima) and type of music alteration on how participants responded to music.

## Results

First we analysed between-group differences to establish how baseline BP affects physiological responses to music, before examining how each group responded to tempo and loudness features. Baseline-normalised average diastolic and systolic BP were compared across groups to establish whether nominal BP, collected at baseline with a cuff, modulates response to music. Within both H-bBP and N-bBP groups, the difference between average BP values during music pieces was significantly greater than zero (baseline) for both systolic BP (H-bBP = 0.0002; N-bBP: P = 5.16e-14) and diastolic BP (H-bBP: P = 1.06e-05; N-bBP: P = 5.74e-18). Western classical pieces played by expert performers, both computationally altered and in their original form, raised average BP above baseline for both participant groups.

### Between-group effects: differences in response to music and music features

#### Response to music

**Fig. 1:**
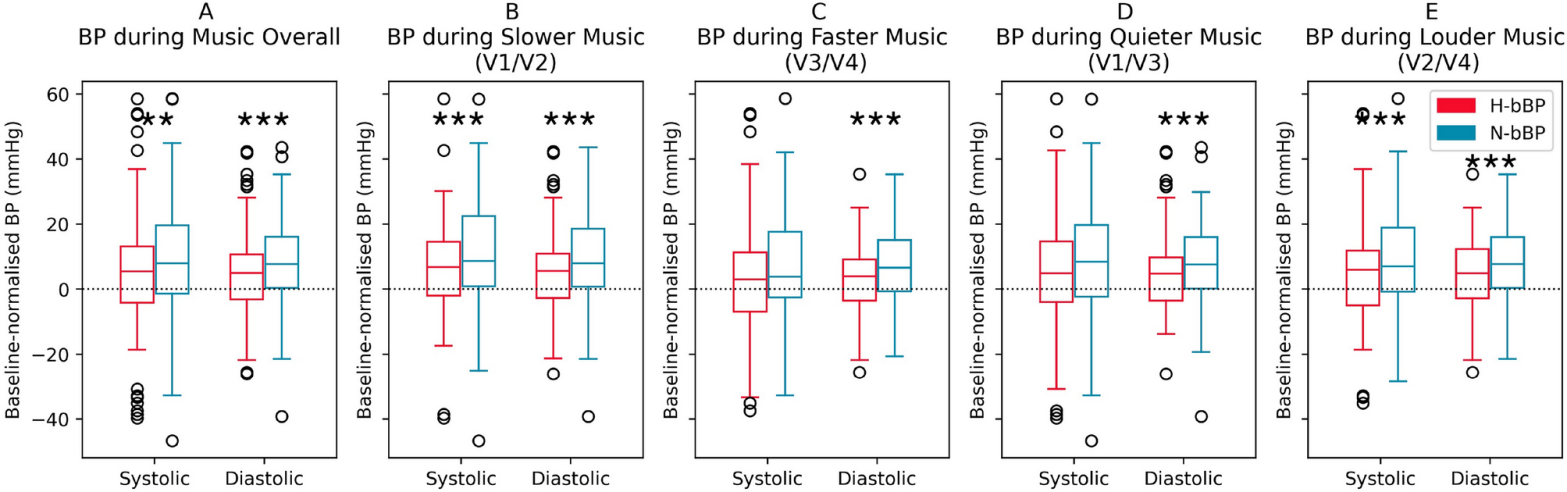
Mean values of normalised systolic and diastolic BP during baseline and music, over all music pieces. Both systolic and diastolic baseline-normalised BP averages are significantly higher for N-bBP participants during music overall, slower music and louder music. During faster and quieter music, only N-bBP’s diastolic BP averages are significantly higher than H-bBP’s. P-values below 0.0083 are indicated with ***.

 The mean increase (reactivity) in systolic and diastolic BP during music (all versions together) was significantly higher for N-bBP than H-bBP participants (mean (95%CI) mmHg): systolic 10.12 (7.70-12.53) vs 3.61 (1.37-5.85), P = 0.00020; diastolic 9.38 (7.58-11.19) vs 3.95 (2.16-5.73), P = 7.25e-05 (Fig. 1A). BP variability, indexed by BP range and the standard deviation of BP, was not significantly different between groups (see Table 3).

#### Tempo and loudness alterations: baseline-comparisons

 Counter-intuitively, systolic and diastolic BP was closest to baseline during the fastest music pieces for both groups. During quieter and sped-up pieces, the difference in systolic BP between groups is no longer significant, though N-bBP’s diastolic BP increase remains significantly higher than H-bBP’s across all alteration types (Table 3). There were no between-group differences in BP variability for any category of music alteration, though within-group analyses revealed another layer of N-bBP sensitivity to music in BP variability. Neither quieter or slower music lowered diastolic or systolic BP for either group below baseline. However, the music stimuli were not selected for their tranquilising properties.

#### Specific tempo and loudness alterations: versions

 There were no significant differences between groups for any of the versions, indicating that the participants groups did not respond differently based on the type of manipulation (see Table 4).

#### Aggregate loudness alterations

 N-bBP participants’ systolic and diastolic BP averages are significantly higher than H-bBP during loud music (V2 + V4; systolic: 10.61 (7.11-14.11) vs 3.07 (0.04-6.10), P = 0.0068; diastolic: 9.37 (6.78-11.95) vs 3.58 (1.03-6.12), P = 0.0075; Fig. 1E). N-bBP participants are reacting more strongly to loud music than H-bBP participants.

#### Aggregate tempo alterations

 However, N-bBP participants’ systolic and diastolic BP is also significantly higher than H-bBP participants’ during slow music (V1 + V2; systolic: 12.43 (9.08-15.79) vs 5.16 (2.03-8.28), P = 0.0021; diastolic: 10.31 (7.65-12.98) vs 5.43 (2.82-8.04), P = 0.0052; Fig. 1B). During faster (V3 + V4) and quieter (V1 + V3) music, only diastolic averages are significantly different between groups: fast: 8.41 (5.99-10.84) vs 2.40 (0.00-4.80), P = 0.0043; quiet: 9.40 (6.86-11.94) vs 4.28 (1.77-6.79), P = 0.0032; Fig. 1C-D).

### Within-group effects: physiological response to music

Music pieces were split into tertiles based on the ranking of their respective Loudness and Tempo indices. In order to compare the impact of different musical feature indices, three sets of tertiles were created based on maxima, standard deviation and mean for both loudness and tempo.

#### Tempo indices

 Whether music pieces are grouped by tempo maxima, averages or standard deviations, we see no within-group differences between slow and medium pieces for either H-bBP or N-bBP participants. H-bBP participants showed fewer within-group differences than N-bBP participants in all tempo indices. H-bBP systolic and diastolic ranges were affected in all tempo indices, with diastolic standard deviation only significantly lower between pieces with medium and fast maxima (Table 5). By contrast, for N-bBP participants all BP variability features (systolic and diastolic ranges and standard deviations) were significantly different across tempo maxima and tempo average tertiles (Tables 5 and 6).

Indexing by tempo standard deviation showed the fewest within-group differences in BP variability for both groups: N-bBP participants had lower diastolic standard deviation, range and systolic range during fast music, while only systolic and diastolic ranges were lower for H-bBP participants (Table 7). Indexing by average tempo and range showed more significant within-group differences.

#### Loudness indices

 There were no within-group differences for either H-bBP or N-bBP participants in response to differing loudness levels in any of the indices studies (Tables 5, 6, 7). N-bBP participants’ BP variability measures varied between versions (Table 8), with both the Kruskal-Wallis and post-hoc Dunn tests finding P < 0.0083. For H-bBP participants, systolic standard deviation was not significantly different between versions, though other variability indices were.

#### Specific tempo and loudness alterations: versions

 There are no significant differences post-hoc between V1 (slow/quiet) and V2 (slow/loud) or V3 (fast/quiet) and V4 (fast/loud) within either participant group. Notably, these pairs are those that maintain the same tempo: V1 and V2 both have the original tempo, while V3 and V4 have accelerated tempo. When only loudness is manipulated, there are no within-group differences. For N-bBP participants, the change between V1/V3 and V2/V3 affected all BP variability features (Table 8). Both participant groups had reduced systolic and diastolic ranges during V4 pieces, which are both faster and louder than V1.

#### Aggregate tempo and loudness alterations: faster or louder

 During sped-up pieces, (V3 + V4: 129.36 bpm (104.45-154.28)) both groups showed significantly reduced BP variability compared to pieces with original tempo (V1 + V2: 63.02 bpm (44.47-81.57)) (P < 0.0083) (Table 9). By contrast, louder pieces (V2 + V4: 17.72 sones (16.15-19.29)) did not reduce BP variables in comparison to quieter pieces (V1 + V3: 11.71 sones (10.22-13.20)) (Table 10). Speeding up musical tempo affected BP variability significantly, while making the music louder did not.

#### BP variability

 Within both groups, BP variability (standard deviation and range) is affected by music features while average BP is not. Fast tempo pieces - whether defined by tempo maximum, average or standard deviation - decreased BP variability, though the effect was more consistent for N-bBP participants. This may be due in part to faster pieces being shorter in duration, leaving less time for BP to vary. Tempo only significantly lowered BP variability above a threshold of $\sim$ 120 bpm. Overall loudness had no significant impact on either participant group, suggesting that global tempo levels have more impact on BP variability than global loudness features. Further, there were no significant differences in BP mean or variability between versions where only loudness was altered. Again, N-bBP participants’ BP variability was more sensitive to versions than H-bBP participants’.

## Discussion

Expressive musical performance had an activating physiological effect: the 10.12 mmHg increase in systolic BP for N-bBP participants is comparable to 10 mmHg increases found during cold pressor tests designed to stress the autonomic system^45^. N-bBP participants’ diastolic and systolic BP rose significantly more than H-bBP participants’. N-bBP participants had higher BP response than H-bBP participants to quietest music, which suggests that greater sensitivity could be a feature of healthy response to music. The greater responsiveness of N-bBP participants to music may reflect their more flexible physiology. H-bBP participants, with their already high baseline BP, may have less capacity to increase their blood pressure.

Alternatively, H-bBP participants could have already been maximally engaged during baseline, so were less activated by the music itself. However, this seems unlikely given both groups were exposed to the same conditions. It is also possible that asking questions of participants between pieces prevented them from relaxing during the music, though both groups were asked questions, so comparisons between- and within-groups are still valid. Only 10% of participants stated they regularly listened to classical music, so the rise in BP could reflect a stress response to an unpleasant experience.

While participants may not be choosing to listen to classical music, they would be familiar with it from film and TV soundtracks, advertisements and even public spaces. The tonal language of Western classical music is shared with pop music, so even without training or active listening, participants would be able to interpret the expressive shape of a classical piece of music. While a formal analysis of the qualitative responses has yet to be conducted, few participants reported an unpleasant experience. The connection between participants’ qualitative and physiological responses will be examined in a separate future analysis focused on individual rather than group-level trends. While the two groups did not differ in overall music sophistication scores, the higher proportion of musicians with >= 4 years of musical training in the H-bBP group could contribute to their lower sensitivity. Classical piano music was selected for this research for its expressive complexity and ability to be played on a reproducing piano. While both groups in this study had the same proportion of classical music fans, it would be valuable to evaluate how manipulated loudness and tempo features impact physiology in other genres as musical preference may impact participant response.

In our music dataset, there was high overlap between tempo features: a piece with high tempo maxima was likely to also have high average tempo, whereas loudness indices were more divergent. Tempo’s greater coherence as a feature may help explain its greater overall impact on BP variability. Our findings highlight the value of musical time structure in the reception of music over its amplitude, consistent with other research on the impact of music tempo^36,37,39,46–49^. Loudness, an obvious acoustic feature, barely impacted the summary statistics for physiological changes. However, acute BP may respond to local shifts in tempo and loudness, so a study of beat-to-beat shifts and musical features – already found to impact musicians’ RR intervals while playing^50^ and listeners respiratory and RR intervals during music^51^ – can inform the design of music therapeutic tools.

While music has been shown to have a positive effect on physiological wellbeing, we found the real-time effect of listening to expressive music led to greater BP elevation particularly in participants with normal baseline blood pressure. Most research aims to reduce BP with music, and only a few report that music raises BP (e.g. Refs.^31,39^). As with music’s excitatory impact on BP, it is surprising to note that both groups showed the lowest BP variability – generally considered a positive physiological indicator – during music computationally altered to be on average twice as fast (range: 1.8 to 3.8). Fast tempo is generally considered to be an excitatory feature in music generated for therapeutics, though these don’t focus on aesthetic or expressive qualities^52^. However, the performer’s expression - the way they convey the piece’s intent and structure and maintain connection to the listener - is at its most appropriate and clear in the original tempo. Listeners’ lower BP variability at high tempo could be due to the mismatch between expression and content, causing listeners to be less engaged and responsive to the piece. Faster music may decouple cardiac response to musical features, or this finding could be due to faster pieces in this study having shorter durations.

Flexibility and responsiveness, a sign of adaptability to both mental and physical challenges, is generally good for the body. BP variability has been found to be a negative indicator, but laboratory response to stressors have been found to be uncorrelated with 24h BPV recordings, raising questions about the usefulness of acute lab-based measurements of BPV but also opening the possibility that acute, short-term high BPV may not be correlated with longer-term high BPV associated with negative health outcomes^9^. However, even at the very short-term beat-to-beat level abnormally high BPV distinguished unhealthy patients from healthy patients with the same mean BP^53,54^. We found more flexibility and variability in acute BP measurements for healthier participants, and greater BP elevation, which together suggest beat-to-beat BP variability might be an indicator of good health in some situations.

An intriguing possibility is that normotensives’ rise in BP is a healthy response to the experience of listening to expressive music. Perhaps, as in physical exercise, healthy individuals’ baroreflexes reset to tolerate higher BP levels as a homeostatic reaction to perturbations caused by the music. The analysis presented here is based solely on summary statistics and may overlook short time-sensitive BP features that help explain the mechanisms driving acute physiological response to music. For example, sudden spikes in diastolic BP have been hypothesised to trigger BP suppressant branches of the autonomic nervous system to alter BP levels and variability^55^. Hypertension is associated with dramatic spikes in response to stressors^56^, so participants with high baseline BP might be prone to more sudden spikes in BP that in turn repeatedly trigger suppressant responses to musical activation, leading to lower BP average response, but a greater range. However, we did not find significantly different BP ranges between groups.

The cardiac control system responds to mental and physical challenges. It buffers the body’s fluctuations in blood pressure that occur in response to challenge via the baroreflexes^57^. Diastolic and systolic BP variability can carry different diagnostic associations^58^. Low beat-to-beat diastolic BP variability has been associated with high heart rate variability, potentially through a healthy, dampening baroreflex response to high systolic BP^57^. Nonetheless, participants in BPV studies exploring its diagnostic value are often cardiovascular patients or otherwise unwell. Hence, these studies may be skewed towards diseased individuals. One example of a study on healthy individuals considered responses to a cold-pressor test designed to stress the body and raise BP^45^. While this study concludes high variability is associated with stiffer arteries, BPV also rose temporarily as part of a healthy response to a physical challenge. Thus, in the short term, a raised BPV may not always be associated with a poor prognosis and future research could examine BPV recovery after stressors.

Systolic standard deviation was the least responsive to music features, particularly for H-bBP participants, while diastolic range was most consistently affected by music features. Systolic and diastolic range were the most sensitive physiological measures to musical features across all participant groups. The sensitivity of range may be due to the indices’ inclusion of outlier BP values. The difference between H-bBP and N-bBP participants was most evident with systolic standard deviation, which was never significantly altered within the H-bBP group by any musical features. The greater sensitivity of diastolic measures across both participant groups – diastolic standard deviation and range to music feature tertiles, average diastolic BP to alteration type – highlights its value in assessing BP response and reactivity. Muscle sympathetic nerve activity (MSNA), an index of sympathetic activation that is highly variable among individuals, occurs primarily during diastole. MSNA has a close negative correlation to diastolic BP: higher diastolic BP means fewer MSNA bursts, thus lower vasoconstriction signals^4,59^. Diastolic BP drops during purposeful muscle relaxation^60^, so the sensitivity of diastolic BP may reflect the measure’s sensitivity to fluctuations in muscle tension associated with stress^61^.

### Conclusions

N-bBP participants’ BP is more responsive to music: music raises baseline-normalised average BP more for N-bBP participants than for H-bBP participants. N-bBP’s BP variability indices (diastolic and systolic ranges and standard deviations) are more consistently lowered in response to sped-up music. Not only does music impact average BP, but responsiveness to music is affected by baseline BP. We found that N-bBP participants not only respond physiologically more to music, but also show more differentiated responses to music features than H-bBP participants.

Unexpectedly, the rapid, rippling-effect of sped-up music led to decreased BP variability for both groups and N-bBP participants’ showed higher BP levels. Our finding that slow music — in our dataset, these are largely the original tempi (unaltered) chosen by the performers — raises BP more is at odds with research on entrainment between tempo and BP and suggests that music pieces in their original expressive performance have a greater physiological effect. In both groups, the fastest music pieces were associated with decreased BPV compared to the slow and medium pieces, calling into question the truism that slow music is relaxing. These findings also invite further research into the relationship between high BPV in response to acute events and high BPV in the long-term, known to be associated with negative health outcomes.

Expressive music is a dynamic stimuli that could encourage variability and positively exercise our autonomic responsiveness. Our research shows that expressive music has a strong impact on blood pressure levels and that musical features interact with participant physiology to alter variability. Longitudinal research on the impact of music, paired with detailed analysis of musical features and acute physiological response, will help bridge the gap between apparently adverse acute BP responses during music-listening and its long-term positive impact.

**Table 3:** N-bBP group’s higher responsiveness compared to H-bBP’s are evident in systolic and diastolic averages, during music, during louder music, during slowest music, and only in diastolic BP averages for quietest and fastest music.

	H-bBP	N-bBP	MWU test
During music (n = 180)			
Systolic BP (mean)	3.61 (1.37-5.85)	10.12 (7.70-12.53)	P = 0.00020**
Diastolic BP (mean)	3.95 (2.16-5.73)	9.38 (7.58-11.19)	P = 7.25e-05**
Systolic standard deviation	5.02 (4.70-5.33)	5.19 (4.84-5.55)	P = 0.87
Diastolic standard deviation	3.86 (3.64-4.07)	3.79 (3.54-4.04)	P = 0.27
Systolic range	20.79 (19.40-22.19)	21.18 (19.80-22.57)	P = 0.81
Diastolic range	16.47 (15.51-17.42)	16.03 (14.99-17.07)	P = 0.38
During quietest (n = 88)			
Systolic BP (mean)	4.09 (0.83-7.36)	9.67 (6.33-13.01)	P = 0.01
Diastolic BP (mean)	4.28 (1.77-6.79)	9.40 (6.86-11.94)	P = 0.0032**
Systolic standard deviation	5.08 (4.67-5.49)	5.05 (4.59-5.51)	P = 0.44
Diastolic standard deviation	3.81 (3.53-4.10)	3.75 (3.45-4.05)	P = 0.41
Systolic range	21.35 (19.59-23.11)	21.09 (19.17-23.02)	P = 0.52
Diastolic range	16.56 (15.27-17.85)	16.21 (14.87-17.55)	P = 0.64
During loudest (n = 92)			
Systolic BP (mean)	3.07 (0.04-6.10)	10.61 (7.11-14.11)	P = 0.0068**
Diastolic BP (mean)	3.58 (1.03-6.12)	9.37 (6.78-11.95)	P = 0.0075**
Systolic standard deviation	4.95 (4.47-5.44)	5.35 (4.80-5.90)	P = 0.27
Diastolic standard deviation	3.90 (3.57-4.23)	3.84 (3.42-4.25)	P = 0.48
Systolic range	20.18 (17.96-22.40)	21.28 (19.27-23.29)	P = 0.28
Diastolic range	16.36 (14.94-17.79)	15.84 (14.22-17.45)	P = 0.43
During slowest (n = 99)			
Systolic BP (mean)	5.16 (2.03-8.28)	12.43 (9.08-15.79)	P = 0.0021**
Diastolic BP (mean)	5.43 (2.82-8.04)	10.31 (7.65-12.98)	P = 0.0052**
Systolic standard deviation	5.47 (4.97-5.98)	5.88 (5.35-6.41)	P = 0.33
Diastolic standard deviation	4.31 (3.97-4.65)	4.29 (3.96-4.62)	P = 0.86
Systolic range	23.85 (21.58-26.11)	25.00 (23.04-26.96)	P = 0.26
Diastolic range	19.17 (17.74-20.61)	18.88 (17.57-20.19)	P = 0.88
During fastest (n = 81)			
Systolic BP (mean)	1.99 (-1.19-5.18)	7.69 (4.27-11.11)	P = 0.03
Diastolic BP (mean)	2.40 (-0.00-4.80)	8.41 (5.99-10.84)	P = 0.0043**
Systolic standard deviation	4.54 (4.20-4.89)	4.47 (4.05-4.89)	P = 0.37
Diastolic standard deviation	3.38 (3.15-3.61)	3.27 (2.92-3.62)	P = 0.14
Systolic range	17.60 (16.28-18.92)	17.19 (15.60-18.78)	P = 0.31
Diastolic range	13.64 (12.69-14.58)	13.06 (11.67-14.45)	P = 0.11

Significant values are in bold.

**Table 4:** There were no significant differences between groups during any version independently, suggesting that no particular combination of tempo and loudness had a consistently different impact on N-bBP vs H-bBP participants.

	H-bBP	N-bBP	MWU test
V1 (n = 51)			
Systolic BP (mean)	5.86 (0.94-10.78)	13.09 (8.31-17.88)	P = 0.02
Diastolic BP (mean)	6.66 (2.89-10.44)	10.57 (6.84-14.31)	P = 0.06
Systolic standard deviation	5.47 (4.86-6.08)	5.88 (5.25-6.52)	P = 0.62
Diastolic standard deviation	4.19 (3.78-4.61)	4.39 (3.98-4.80)	P = 0.55
Systolic range	24.06 (21.46-26.65)	25.61 (23.02-28.19)	P = 0.47
Diastolic range	18.88 (17.09-20.67)	19.63 (17.92-21.33)	P = 0.45
V2 (n = 48)			
Systolic BP (mean)	4.28 (0.80-7.76)	11.61 (6.96-16.27)	P = 0.06
Diastolic BP (mean)	3.89 (0.40-7.38)	9.99 (6.16-13.81)	P = 0.03
Systolic standard deviation	5.48 (4.63-6.34)	5.88 (4.99-6.77)	P = 0.40
Diastolic standard deviation	4.45 (3.91-5.00)	4.16 (3.62-4.70)	P = 0.37
Systolic range	23.59 (19.62-27.55)	24.24 (21.23-27.26)	P = 0.40
Diastolic range	19.54 (17.18-21.89)	17.95 (15.94-19.96)	P = 0.29
V3 (n = 37)			
Systolic BP (mean)	2.04 (-2.08-6.17)	5.71 (1.33-10.09)	P = 0.32
Diastolic BP (mean)	1.51 (-1.55-4.56)	8.04 (4.67-11.41)	P = 0.03
Systolic standard deviation	4.62 (4.11-5.13)	4.09 (3.55-4.62)	P = 0.09
Diastolic standard deviation	3.37 (3.03-3.71)	3.01 (2.68-3.33)	P = 0.07
Systolic range	18.20 (16.23-20.18)	15.86 (13.89-17.84)	P = 0.06
Diastolic range	13.86 (12.33-15.40)	12.25 (10.83-13.67)	P = 0.10
V4 (n = 44)			
Systolic BP (mean)	1.94 (-2.95-6.84)	9.67 (4.44-14.90)	P = 0.04
Diastolic BP (mean)	3.29 (-0.43-7.00)	8.79 (5.26-12.31)	P = 0.09
Systolic standard deviation	4.46 (4.00-4.92)	4.86 (4.23-5.50)	P = 0.53
Diastolic standard deviation	3.39 (3.07-3.71)	3.53 (2.92-4.14)	P = 0.81
Systolic range	17.00 (15.24-18.76)	18.52 (16.07-20.97)	P = 0.67
Diastolic range	13.41 (12.28-14.54)	13.86 (11.48-16.25)	P = 0.58

**Table 5:** Both H-bBP and N-bBP showed significant within-group drops in BP variability in response to raised tempo maxima, but not to raised levels of loudness maxima (Kruskal-Wallis test, followed by a post-hoc Dunn test).

	Tempo maxima subgroups				Subgroup comparisons		
	Slow (n = 52)	Med (m = 66)	Fast (o = 62)	KW	Slow/fast	Slow/med	Med/fast
H-bBP							
Sys (mean)	2.93 (-0.57-6.43)	3.64 (-0.69-7.97)	4.15 (0.58-7.71)	P = 0.99	N/A	N/A	N/A
Dia (mean)	3.62 (0.41-6.84)	4.29 (0.91-7.68)	3.85 (1.23-6.46)	P = 0.86	N/A	N/A	N/A
Sys (std dev)	5.31 (4.65-5.97)	5.34 (4.78-5.90)	4.43 (4.04-4.81)	P = 0.03	N/A	N/A	N/A
Dia (std dev)	4.13 (3.70-4.56)	4.10 (3.73-4.48)	3.36 (3.07-3.65)	P = 0.004**	P = 0.017	P = 0.83	P = 0.008**
Sys (range)	23.02 (19.83-26.21)	22.52 (20.23-24.80)	17.10 (15.57-18.62)	P = 0.0003**	P = 0.002**	P = 0.91	P = 0.0009**
Dia (range)	18.31 (16.43-20.19)	17.77 (16.17-19.38)	13.53 (12.28-14.78)	P = 2.28e-05**	P = 0.0002**	P = 0.79	P = 0.0002**
N-bBP							
Sys (mean)	9.76 (5.31-14.21)	12.65 (8.68-16.62)	7.71 (3.59-11.84)	P = 0.18	N/A	N/A	N/A
Dia (mean)	7.33 (3.86-10.80)	11.64 (8.56-14.72)	8.71 (5.87-11.55)	P = 0.28	N/A	N/A	N/A
Sys (std dev)	5.64 (4.98-6.30)	5.73 (5.10-6.35)	4.25 (3.76-4.73)	P = 7.9e-05**	P = 0.0009**	P = 0.84	P = 0.0002**
Dia (std dev)	4.29 (3.71-4.86)	4.11 (3.70-4.51)	3.04 (2.78-3.29)	P = 8.7e-06**	P = 0.0001**	P = 0.99	P = 0.00006**
Sys (range)	23.71 (21.21-26.21)	24.06 (21.61-26.51)	16.00 (14.38-17.62)	P = 7.8e-08**	P = 0.000003**	P = 0.95	P = 0.000001**
Dia (range)	18.69 (16.51-20.87)	17.62 (15.98-19.26)	12.11 (10.96-13.27)	P = 1.5e-08**	P = 2.9 E-07**	P = 0.5145	P= 0.000001**

	Loudness maxima subgroups				Subgroup comparisons		
	Quiet (n = 53)	Med (m = 54)	Loud (o = 73)	KW	Quiet/loud	Quiet/med	Med/loud
H-bBP							
Sys (mean)	4.50 (0.22-8.79)	0.61 (-2.99-4.22)	5.18 (1.52-8.85)	P = 0.36	N/A	N/A	N/A
Dia (mean)	4.13 (1.29-6.98)	1.88 (-1.32-5.09)	5.34 (2.27-8.40)	P = 0.14	N/A	N/A	N/A
Sys (std dev)	5.18 (4.56-5.79)	5.01 (4.32-5.69)	4.91 (4.52-5.30)	P = 0.74	N/A	N/A	N/A
Dia (std dev)	3.80 (3.38-4.22)	3.86 (3.43-4.29)	3.89 (3.59-4.20)	P = 0.71	N/A	N/A	N/A
Sys (range)	21.49 (18.94-24.04)	20.61 (17.36-23.86)	20.42 (18.74-22.11)	P = 0.57	N/A	N/A	N/A
Dia (range)	16.38 (14.58-18.17)	16.37 (14.46-18.28)	16.60 (15.21-17.99)	P = 0.74	N/A	N/A	N/A
N-bBP							
Sys (mean)	9.62 (5.07-14.17)	7.31 (3.46-11.17)	12.55 (8.54-16.56)	P = 0.15	N/A	N/A	N/A
Dia (mean)	8.82 (5.43-12.21)	8.33 (4.99-11.68)	10.57 (7.78-13.36)	P = 0.61	N/A	N/A	N/A
Sys (std dev)	5.14 (4.48-5.79)	4.59 (4.15-5.04)	5.68 (5.04-6.32)	P = 0.07	N/A	N/A	N/A
Dia (std dev)	3.70 (3.27-4.12)	3.64 (3.13-4.16)	3.97 (3.58-4.35)	P = 0.23	N/A	N/A	N/A
Sys (range)	20.77 (18.21-23.34)	18.85 (16.93-20.77)	23.21 (20.78-25.63)	P = 0.07	N/A	N/A	N/A
Dia (range)	15.74 (14.00-17.47)	15.30 (13.23-17.36)	16.79 (15.16-18.43)	P = 0.34	N/A	N/A	N/A

All BP variability measures were significantly lower in post-hoc tests for N-bBP participants, but only diastolic standard deviation, systolic and diastolic ranges were lower for H-bBP participants. Significant values are in bold.

**Table 6:** Both H-bBP and N-bBP showed significant within-group drops in BP variability response to higher average tempo, but not to higher average loudness (Kruskal-Wallis test, followed by a post-hoc Dunn test).

	Tempo average subgroups				Subgroup comparisons		
	Slow (n = 63)	Med (m = 55)	Fast (o = 62)	KW	Slow/fast	Slow/med	Med/fast
H-bBP							
Sys (mean)	4.21 (0.82-7.59)	2.32 (-2.45-7.09)	4.15 (0.58-7.71)	P = 0.86	N/A	N/A	N/A
Dia (mean)	4.34 (1.45-7.23)	3.61 (-0.24-7.46)	3.85 (1.23-6.46)	P = 0.99	N/A	N/A	N/A
Sys (std dev)	5.37 (4.71-6.02)	5.29 (4.76-5.81)	4.43 (4.04-4.81)	P = 0.03	N/A	N/A	N/A
Dia (std dev)	4.18 (3.76-4.60)	4.04 (3.67-4.41)	3.36 (3.07-3.65)	P = 0.004**	P = 0.012	P = 0.96	P = 0.012
Sys (range)	23.33 (20.36-26.31)	22.05 (19.84-24.27)	17.10 (15.57-18.62)	P = 0.0003**	P = 0.001**	P = 0.92	P = 0.002**
Dia (range)	18.76 (16.99-20.54)	17.15 (15.52-18.77)	13.53 (12.28-14.78)	P = 1.4e-05**	P = 0.000017**	P = 0.31	P = 0.0015**
N-bBP							
Sys (mean)	9.93 (6.03-13.84)	13.03 (8.52-17.54)	7.71 (3.59-11.84)	P = 0.18	N/A	N/A	N/A
Dia (mean)	8.38 (5.36-11.39)	11.30 (7.72-14.88)	8.71 (5.87-11.55)	P = 0.63	N/A	N/A	N/A
Sys (std dev)	5.66 (5.05-6.27)	5.73 (5.05-6.40)	4.25 (3.76-4.73)	P = 7.73e-05**	P = 0.0005**	P = 0.76	P = 0.0004**
Dia (std dev)	4.27 (3.78-4.77)	4.08 (3.63-4.54)	3.04 (2.78-3.29)	P = 8.5e-06**	P = 0.00005**	P = 0.84	P = 0.00013**
Sys (range)	23.94 (21.67-26.21)	23.87 (21.14-26.60)	16.00 (14.38-17.62)	P = 7.40e-08**	P = 6.8e-07**	P = 0.73	P = 6.1e-06**
Dia (range)	18.92 (16.95-20.89)	17.15 (15.43-18.86)	12.11 (10.96-13.27)	P = 1.0e-08**	P = 2.4e-08**	P = 0.26	P= 0.000017**

	Loudness average sungroups				Sungroup comparsion		
	Quiet (n = 56)	Med (m = 67)	Loud (o = 57)	KW	Quiet/loud	Quiet/med	Med/loud
H-bBP							
Sys (mean)	3.46 (-0.98-7.90)	4.16 (0.37-7.95)	3.16 (-0.41-6.74)	P = 0.90	N/A	N/A	N/A
Dia (mean)	3.88 (0.86-6.90)	4.93 (1.67-8.18)	3.00 (0.10-5.91)	P = 0.83	N/A	N/A	N/A
Sys (std dev)	5.11 (4.48-5.74)	4.91 (4.52-5.31)	5.05 (4.44-5.67)	P = 0.76	N/A	N/A	N/A
Dia (std dev)	3.75 (3.30-4.20)	3.86 (3.58-4.14)	3.93 (3.53-4.34)	P = 0.49	N/A	N/A	N/A
Sys (range)	21.47 (18.80-24.14)	20.65 (18.88-22.42)	20.43 (17.62-23.24)	P = 0.42	N/A	N/A	N/A
Dia (range)	16.49 (14.60-18.38)	16.59 (15.23-17.95)	16.32 (14.55-18.10)	P = 0.62	N/A	N/A	N/A
N-bBP							
Sys (mean)	10.98 (6.36-15.59)	10.45 (6.48-14.41)	9.13 (5.04-13.22)	P = 0.44	N/A	N/A	N/A
Dia (mean)	9.87 (6.24-13.50)	9.53 (6.44-12.62)	8.87 (6.05-11.70)	P = 0.78	N/A	N/A	N/A
Sys (std dev)	5.33 (4.65-6.01)	5.24 (4.60-5.88)	5.04 (4.51-5.58)	P = 0.92	N/A	N/A	N/A
Dia (std dev)	3.85 (3.42-4.27)	3.90 (3.47-4.34)	3.63 (3.19-4.07)	P = 0.71	N/A	N/A	N/A
Sys (range)	22.12 (19.45-24.79)	21.70 (19.14-24.26)	19.95 (17.97-21.94)	P = 0.52	N/A	N/A	N/A
Dia (range)	16.61 (14.78-18.45)	16.71 (15.01-18.41)	14.91 (13.07-16.75)	P = 0.11	N/A	N/A	N/A

All BP variability measures dropped significantly for N-bBP participants, but only systolic and diastolic ranges for H-bBP participants according to post-hoc tests. Significant values are in bold.

**Table 7:** Both H-bBP and N-bBP showed significant within-group drops of systolic and diastolic BP variation in response to higher standard deviation of tempo, but not to different levels of standard deviation in loudness (Kruskal-Wallis test, followed by a post-hoc Dunn test).

	Tempo std dev subgroups				Subgroup comparisons		
	Slow (n = 52)	Med (m = 69)	Fast (o = 59)	KW	Slow/fast	Slow/med	Med/fast
H-bBP							
Sys (mean)	2.93 (-0.57-6.43)	4.90 (0.77-9.02)	2.70 (-1.05-6.45)	P = 0.60	N/A	N/A	N/A
Dia (mean)	3.62 (0.41-6.84)	4.98 (1.82-8.15)	3.02 (0.18-5.86)	P = 0.51	N/A	N/A	N/A
Sys (std dev)	5.31 (4.65-5.97)	5.26 (4.71-5.80)	4.48 (4.08-4.88)	P = 0.07	N/A	N/A	N/A
Dia (std dev)	4.13 (3.70-4.56)	4.01 (3.65-4.37)	3.44 (3.13-3.75)	P = 0.020	N/A	N/A	N/A
Sys (range)	23.02 (19.83-26.21)	22.12 (19.88-24.35)	17.29 (15.71-18.87)	P = 0.002**	P = 0.0052**	P = 0.86	P = 0.0052**
Dia (range)	18.31 (16.43-20.19)	17.39 (15.80-18.98)	13.76 (12.47-15.06)	P = 0.0002**	P = 0.00048**	P = 0.55	P = 0.0012**
N-bBP							
Sys (mean)	9.76 (5.31-14.21)	10.88 (6.95-14.80)	9.54 (5.27-13.81)	P = 0.87	N/A	N/A	N/A
Dia (mean)	7.33 (3.86-10.80)	10.41 (7.49-13.33)	10.00 (6.94-13.06)	P = 0.61	N/A	N/A	N/A
Sys (std dev)	5.64 (4.98-6.30)	5.46 (4.86-6.07)	4.48 (3.95-5.01)	P = 0.006**	P = 0.011	P = 0.63	P = 0.017
Dia (std dev)	4.29 (3.71-4.86)	3.94 (3.54-4.34)	3.17 (2.89-3.45)	**P = 0.002**	P = 0.0025**	P = 0.46	P = 0.0097**
Sys (range)	23.71 (21.21-26.21)	23.03 (20.59-25.47)	16.80 (15.00-18.59)	**P = 2.5e-05**	P = 0.000078**	P = 0.45	P = 0.00038**
Dia (range)	18.69 (16.51-20.87)	16.88 (15.25-18.52)	12.69 (11.42-13.97)	**P = 5.5e-06**	P = 0.000008**	P = 0.17	P = 0.00059**

	Loudness std dev subgroups				Subgroup comparisons		
	Quiet (n = 58)	Med (m = 60)	Loud (o = 62)	KW	Quiet/loud	Quiet/med	Med/loud
H-bBP							
Sys (mean)	5.86 (2.01-9.71)	2.20 (-1.72-6.12)	2.87 (-0.97-6.71)	P = 0.37	N/A	N/A	N/A
Dia (mean)	5.05 (2.18-7.93)	3.74 (0.35-7.13)	3.11 (0.10-6.12)	P = 0.72	N/A	N/A	N/A
Sys (std dev)	5.29 (4.71-5.87)	4.63 (4.25-5.01)	5.15 (4.51-5.78)	P = 0.36	N/A	N/A	N/A
Dia (std dev)	3.85 (3.44-4.26)	3.75 (3.49-4.01)	3.96 (3.53-4.39)	P = 0.94	N/A	N/A	N/A
Sys (range)	22.24 (19.78-24.71)	19.32 (17.57-21.06)	20.87 (18.00-23.75)	P = 0.27	N/A	N/A	N/A
Dia (range)	16.83 (15.06-18.60)	16.23 (14.88-17.59)	16.35 (14.53-18.18)	P = 0.75	N/A	N/A	N/A
N-bBP							
Sys (mean)	9.26 (4.94-13.57)	8.76 (4.63-12.90)	12.23 (8.13-16.33)	P = 0.66	N/A	N/A	N/A
Dia (mean)	8.13 (4.88-11.39)	10.09 (7.03-13.14)	9.88 (6.76-12.99)	P = 0.90	N/A	N/A	N/A
Sys (std dev)	5.20 (4.56-5.83)	5.30 (4.69-5.92)	5.08 (4.49-5.68)	P = 0.78	N/A	N/A	N/A
Dia (std dev)	3.72 (3.33-4.10)	4.13 (3.56-4.70)	3.53 (3.23-3.82)	P = 0.53	N/A	N/A	N/A
Sys (range)	21.29 (18.72-23.87)	22.45 (19.95-24.95)	19.85 (17.72-21.99)	P = 0.37	N/A	N/A	N/A
Dia (range)	16.07 (14.36-17.78)	17.85 (15.60-20.10)	14.24 (13.01-15.48)	P = 0.08	N/A	N/A	N/A

Diastolic standard deviation, systolic and diastolic ranges were significantly lower between Slow/Fast and Med/Fast for N-bBP participants in post-hoc tests, but only systolic and diastolic ranges were different for H-bBP participants. Significant values are in bold.

**Table 8:** N-bBP participants’ BP variability drops more consistently in response to tempo manipulations than H-bBP participants’.

	Versions				
	V1 (n = 51)	V2 (LOUDER, m = 41)	V3 (FASTER, o = 41)	V4 (BOTH, q = 40)	Kruskal-Wallis Test
H-bBP					
Sys (mean)	5.86 (0.94-10.78)	4.28 (0.80-7.76)	1.71 (-2.88-6.31)	2.81 (-2.27-7.90)	P = 0.62
Dia (mean)	6.66 (2.89-10.44)	3.89 (0.40-7.38)	1.09 (-2.30-4.47)	3.97 (0.08-7.87)	P = 0.30
Sys (std dev)	5.47 (4.86-6.08)	5.48 (4.63-6.34)	4.59 (4.02-5.16)	4.37 (3.89-4.85)	P = 0.010
Dia (std dev)	4.19 (3.78-4.61)	4.45 (3.91-5.00)	3.44 (3.07-3.80)	3.29 (2.98-3.59)	P = 0.00042**
Sys (range)	24.06 (21.46-26.65)	23.59 (19.62-27.55)	18.27 (16.06-20.48)	16.77 (14.90-18.65)	P = 3.27e-05**
Dia (range)	18.88 (17.09-20.67)	19.54 (17.18-21.89)	14.16 (12.50-15.83)	13.22 (12.08-14.37)	P = 4.41e-07**
N-bBP					
Sys (mean)	13.09 (8.31-17.88)	11.61 (6.96-16.27)	4.93 (0.67-9.19)	8.54 (3.10-13.97)	P = 0.04
Dia (mean)	10.57 (6.84-14.31)	9.99 (6.16-13.81)	7.68 (4.12-11.25)	8.02 (4.54-11.50)	P = 0.50
Sys (std dev)	5.88 (5.25-6.52)	5.88 (4.99-6.77)	4.00 (3.46-4.53)	4.74 (4.10-5.38)	P = 3.30e-05**
Dia (std dev)	4.39 (3.98-4.80)	4.16 (3.62-4.70)	3.06 (2.71-3.41)	3.49 (2.83-4.14)	P = 7.497e-06**
Sys (range)	25.61 (23.02-28.19)	24.24 (21.23-27.26)	15.84 (13.87-17.81)	18.20 (15.70-20.70)	P = 3.034e-08**
Dia (range)	19.63 (17.92-21.33)	17.95 (15.94-19.96)	12.59 (11.14-14.05)	13.57 (11.04-16.11)	P = 1.49e-09**

	Subgroup comparisons (post-hoc Dunn test)					
	V1/V2	V1/V3	V1/V4	V2/V3	V2/V4	V3/V4
H-bBP						
Sys (mean)	N/A	N/A	N/A	N/A	N/A	N/A
Dia (mean)	N/A	N/A	N/A	N/A	N/A	N/A
Sys (std dev)	N/A	N/A	N/A	N/A	N/A	N/A
Dia (std dev)	P = 1.00	P = 0.028	P = 0.0058**	P = 0.026	P = 0.0052**	P = 1.00
Sys (range)	P = 0.59	P = 0.0051**	P = 0.000093**	P = 0.093	P = 0.0073**	P = 0.58
Dia (range)	P = 1.00	P = 0.00082**	P = 0.000080**	P = 0.00082**	P = 0.00011**	P = 1.00
N-bBP						
Sys (mean)	N/A	N/A	N/A	N/A	N/A	N/A
Dia (mean)	N/A	N/A	N/A	N/A	N/A	N/A
Sys (std dev)	P = 0.80	P = 0.00011**	P = 0.046	P = 0.00060**	P = 0. 17	P = 0.17
Dia (std dev)	P = 0.057	P = 0.000041**	P = 0.0023**	P = 0.0022**	P = 0.039	P = 0.57
Sys (range)	P = 0.51	P = 0.000001**	P = 0.00020**	P = 0.000061**	P = 0.0036**	p = 0.46
Dia (range)	p = 0.40	P = 5.28e-07**	P = 1.05e-06**	P = 0.00037**	P = 0.00060**	p = 0.79

Also, N-bBP participants show more comprehensive shifts down of both range and standard deviation for systolic and diastolic BP. Debussy Versions 3 and 4 were excluded from this analysis (see Music Stimuli section for further detail). Significant values are in bold.

**Table 9:** Both H-bBP and N-bBP participants showed significantly lower systolic and diastolic BP standard deviations and ranges during faster music compared to slower.

Tempo alteration (within group)	Slow (n = 99)	Fast (m = 81)	MWU test
H-bBP			
Systolic BP (mean)	5.16 (2.03-8.28)	1.99 (-1.19-5.18)	P = 0.12
Diastolic BP (mean)	5.43 (2.82-8.04)	2.40 (-0.00-4.80)	P = 0.20
Systolic standard deviation	5.47 (4.97-5.98)	4.54 (4.20-4.89)	P = 0.0029**
Diastolic standard deviation	4.31 (3.97-4.65)	3.38 (3.15-3.61)	P = 2.78E-05**
Systolic range	23.85 (21.58-26.11)	17.60 (16.28-18.92)	P = 3.25E-06**
Diastolic range	19.17 (17.74-20.61)	13.64 (12.69-14.58)	P = 5.00E-09**
N-bBP			
Systolic BP (mean)	12.43 (9.08-15.79)	7.69 (4.27-11.11)	P = 0.025
Diastolic BP (mean)	10.31 (7.65-12.98)	8.41 (5.99-10.84)	P = 0.22
Systolic standard deviation	5.88 (5.35-6.41)	4.47 (4.05-4.89)	P = 1.11E-05**
Diastolic standard deviation	4.29 (3.96-4.62)	3.27 (2.92-3.62)	P = 3.59E-07**
Systolic range	25.00 (23.04-26.96)	17.19 (15.60-18.78)	P = 1.46E-09**
Diastolic range	18.88 (17.57-20.19)	13.06 (11.67-14.45)	P = 4.96E-11**

Significant values are in bold.

**Table 10:** Neither H-bBP and N-bBP participants showed significant differences in BP averages or variability in response to loudness alteration.

Loudness alteration (within group)	Quiet (n = 88)	Loud (m = 92)	MWU test
H-bBP			
Systolic BP (mean)	4.09 (0.83-7.36)	3.07 (0.04-6.10)	P = 0.96
Diastolic BP (mean)	4.28 (1.77-6.79)	3.58 (1.03-6.12)	P = 0.88
Systolic standard deviation	5.08 (4.67-5.49)	4.95 (4.47-5.44)	P = 0.24
Diastolic standard deviation	3.81 (3.53-4.10)	3.90 (3.57-4.23)	P = 0.93
Systolic range	21.35 (19.59-23.11)	20.18 (17.96-22.40)	P = 0.12
Diastolic range	16.56 (15.27-17.85)	16.36 (14.94-17.79)	P = 0.69
N-bBP			
Systolic BP (mean)	9.67 (6.33-13.01)	10.61 (7.11-14.11)	P = 0.87
Diastolic BP (mean)	9.40 (6.86-11.94)	9.37 (6.78-11.95)	P = 0.99
Systolic standard deviation	5.05 (4.59-5.51)	5.35 (4.80-5.90)	P = 0.35
Diastolic standard deviation	3.75 (3.45-4.05)	3.84 (3.42-4.25)	P = 0.83
Systolic range	21.09 (19.17-23.02)	21.28 (19.27-23.29)	P = 0.78
Diastolic range	16.21 (14.87-17.55)	15.84 (14.22-17.45)	P = 0.50

## Data Availability

The datasets generated and/or analysed during the current study are not publicly available as they are part of a larger planned data publication but are available from the corresponding author on reasonable request.
